# Clinical outcomes of baricitinib in patients with systemic lupus erythematosus: Pooled analysis of SLE-BRAVE-I and SLE-BRAVE-II trials

**DOI:** 10.1371/journal.pone.0320179

**Published:** 2025-04-30

**Authors:** Juntao Yin, Yantao Hou, Chaoyang Wang, Changjiang Qin

**Affiliations:** 1 Department of Pharmacy, Huaihe Hospital, Henan University, Kaifeng, China; 2 College of New Energy and Intelligent Vehicles, Henan Technical Institute, Zhengzhou, China; 3 Department of General Surgery, Huaihe Hospital, Henan University, Kaifeng, China; University of Colombo Faculty of Medicine, SRI LANKA

## Abstract

**Background:**

Baricitinib is an oral selective inhibitor of Janus kinase 1/2 that has achieved clinically meaningful outcomes for systemic lupus erythematosus (SLE). Only two phase 3 randomized clinical trials (SLE-BRAVE-I and SLE-BRAVE-II) have been completed, but their conclusions were inconsistent. We aimed to determine the efficacy and safety of once-daily oral baricitinib 4 mg or 2 mg treatment versus placebo in participants with active SLE.

**Methods:**

SLE-BRAVE-I and SLE-BRAVE-II are two multicenter, placebo-controlled, double-blind, phase 3 randomized clinical trials with follow-up to 52 weeks. At baseline, 1535 patients (aged ≥18 years) with active SLE (excluding those with central nervous system and severe active renal disease) receiving stable background therapy were randomly assigned 1:1:1 to baricitinib 4 mg, baricitinib 2 mg, or placebo once daily for 52 weeks. The primary endpoint was the proportion of patients with an SLE Responder Index (SRI)-4 response at week 52 in the baricitinib 4 mg treatment group compared with placebo.

**Results:**

There was no difference in the primary efficacy outcome of the proportion of SRI-4 responders at week 52 between participants who received baricitinib 4mg (263 [52%]; odds ratio (OR) 1.27 [95% CI 0.99, 1.63]), 2 mg (246 [48%]; 1.09 [0.85, 1.39]) and placebo (232 [46%]). However, based on SLE-BRAVE-I and SLE-BRAVE-II studies, baricitinib 4 mg and baricitinib 2 mg were more effective in reducing the disease activity in participants who treated with a glucocorticoid dose at baseline of 10 mg per day or higher of prednisone or equivalent, and baricitinib 4 mg was more effective in reducing the disease activity in participants with highly active disease (SLEDAI-2K score at baseline >10). None of the major secondary endpoints, including time to first severe flare and glucocorticoid tapering, were met. Compared with placebo, baricitinib 4 mg and baricitinib 2 mg in the treatment of SLE did not increase the incidence of treatment-emergent adverse events (TEAEs).

**Conclusions:**

Pooled analysis showed that once-daily oral baricitinib 4 mg or baricitinib 2 mg in addition to SOC did not reduce the overall disease activity compared with placebo although they were safe for SLE. However, baricitinib might be a potential treatment option for SLE in certain subpopulation, such as in participants who received a glucocorticoid dose at baseline of 10 mg per day or higher of prednisone or equivalent (baricitinib 4 mg and baricitinib 2 mg) and in participants with highly active disease (SLEDAI-2K score at baseline >10) (baricitinib 4 mg).

**Trial registration number:**

ClinicalTrials. gov Registry (NCT03616912 and NCT03616964).

## Introduction

Systematic lupus erythematosus (SLE) is a multisystem chronic autoimmune disease with extremely various clinical manifestations, which is characterized by systemic inflammation, excessive production of autoantibodies and aberrant activities of the immune system [1,2].

Different from rheumatoid arthritis (RA) and other diseases [3], the main outcomes of SLE, including mortality, did not improve in the first twenty years of this century [4]. One quarter to one third of patients with SLE show poor disease control [5]. With significant morbidity and mortality, SLE is still one of the main causes of death among young women aged 15–45 [4,6]. The positive results of the phase 3 trials of belimumab and anivolumab [7] have promoted regulatory approval for these drugs and supported the involvement of humoral and innate immunity in the pathogenesis of SLE. Many other studies, however, have yielded negative results, and high medical demand for treatment of SLE still cannot be met [8]. Routine treatment of SLE includes antimalarials (hydroxychloroquine), glucocorticoids (prednisone, methylprednisolone) and immunosuppressants (azathioprine, cyclophosphamide, mycophenolate mofetil, methotrexate, rituximab) [9]. However, the control of disease activity in many patients remains unimproved may result in end-organ damage [5]. In order to meet the medical needs, SLE clinical drug development has never stopped, however, in the past 60 years, only two kinds of new treatment have been approved [10].

Many cytokines, such as interferons (IFNs), B-cell activating factor, interleukin (IL)-6, IL-12, IL-17, IL-23, and tumor necrosis factor (TNF), are associated with the etiology of SLE [11–13]. Moreover, these cytokines activate intracellular signaling pathways by activating Janus kinases (JAKs) [1,14,15]. JAK inhibitors, a new category of drugs, can effectively treat a variety of autoimmune diseases [16]. Baricitinib, a selective JAK1/2 inhibitor, is approved for active RA, alopecia areata (AA), and atopic dermatitis (AD) [17].

A phase Ⅱ study showed that at week 24, once-daily oral baricitinib 4 mg plus standard of care (SOC) was superior to SOC in improving the disease activity of SLE [18]. In addition, baricitinib significantly downregulated expression of anti-dsDNA antibodies and key cytokines among seropositive patients [13,19,20].

The baricitinib phase Ⅲ program in severe SLE included two trials, SLE-BRAVE-I [21] and II [22], in which active SLE participants (aged ≥18 years) were enrolled. However, the conclusions of SLE-BRAVE-I and II were inconsistent in the primary outcome. The primary outcome of SLE-BRAVE-I demonstrated that a significantly higher proportion of participants who received baricitinib 4 mg (142 [57%]; odds ratio (OR) 1.57 [95% CI 1.09, 2.27]), but not baricitinib 2 mg (126 [50%]; OR 1.14; 95% CI 0.79, 1.65), reached SLE Responder Index (SRI)-4 response compared with placebo; while the primary outcome of SLE-BRAVE-Ⅱ demonstrated that no significant difference was observed in the proportion of SRI-4 responders at week 52 between participants who received baricitinib 4mg (121 [47%]; OR 1.07; 95% CI 0.75, 1.53]), 2 mg (120 [46%]; OR 1.05; 95% CI 0.73, 1.50) and placebo (116 [46%]). SRI-4 is a composite responder index based on improvement in disease activity (at least 4 point improvement in SLEDAI-2K score) and without the development of substantial disease activity in new organ systems (no new British Isles Lupus Assessment Group (BILAG) A or > 1 new BILAG B. BILAG A means that the disease is active and requiring treatment, and BILAG B means that the activity has improved and there may be some residual or mild symptoms) or worsening of the overall condition (no worsening in Physician Global Assessment, defined as an increase of ≥ 0.3 points [10 mm] from baseline). Therefore, a pooled analysis of the two available phase Ⅲ randomized controlled trials (RCTs) is needed to investigate the efficacy and safety of baricitinib 4 mg or 2 mg compared with placebo in participants with active SLE.

## Materials and methods

Pooled data are included from the available phase 3 cohorts of SLE-BRAVE-I [21] and II [22], two double-blind RCTs followed up for 52 weeks (data cutoffs of November 1, 2020, and August 26, 2020, respectively). Overall, 1535 patients with SLE (met at least 4 of eleven American College of Rheumatology (ACR) SLE classification criteria, excluding those with central nervous system and severe active renal disease) [23,24] were enrolled in SLE-BRAVE-I and II. At baseline participants with SLE were randomly assigned to receive baricitinib 4 mg, baricitinib 2 mg, or placebo in a 1:1:1 ratio.

Both the SLE-BRAVE-I and II studies were performed based on ethical principles of the Declaration of Helsinki and Good Clinical Practice guidelines, and approval was obtained from the authorized institutional review boards at all study sites. All patients provided written informed consent. Each participating center has obtained ethical approval.

The same study design (S1 Fig in Supporting information file) and data collection between the 2 studies allowed us to pool data from all studies to increase statistical power. CONSORT (Consolidated Standards of Reporting Trials) reporting guidelines was followed in this study.

The primary endpoint was the proportion of participants reaching a SRI-4 response at week 52 in those receiving baricitinib 4 mg plus SOC compared with placebo plus SOC. Major secondary endpoints included the proportion of participants reaching a SRI-4 response at week 24 in those who received baricitinib 4 mg or 2 mg compared with SOC, proportion of patients who reached a lupus low disease activity state (LLDAS) [25] response at week 52, proportion of patients who received ≥7.5 mg prednisone (or equivalent) at baseline able to decrease dose by ≥ 25% to a prednisone equivalent dose of ≤ 7.5 mg per day maintained between week 40 and week 52, time to first severe fl....are over 52 weeks, change from baseline in Worst Pain Numeric Rating Scale (NRS) at week 52, change from baseline in Functional Assessment of Chronic Illness Therapy (FACIT)-Fatigue total score at week 52, and proportion of patients in the baricitinib 2 mg group with an SRI-4 response at week 52. At each study visit, adverse events (AEs) reports were collected. Events were coded according to the Medical Dictionary for Regulatory Activities (version 24.0). AEs of special interest included infections (including tuberculosis, herpes zoster, or opportunistic infections), major adverse cardiovascular events (MACEs), malignancies, hepatic events, and venous thromboembolic events (VTEs). The efficacy measures are detailed in S1 Table.

Descriptive statistics are summarized using nonresponder imputation for missing data. Secondary censoring rule rules out data collected after permanent withdrawal of study drug. Statistical analyses were conducted with SAS (version 9.4 or higher, SAS Institute).

## Results

**Fig 1:**
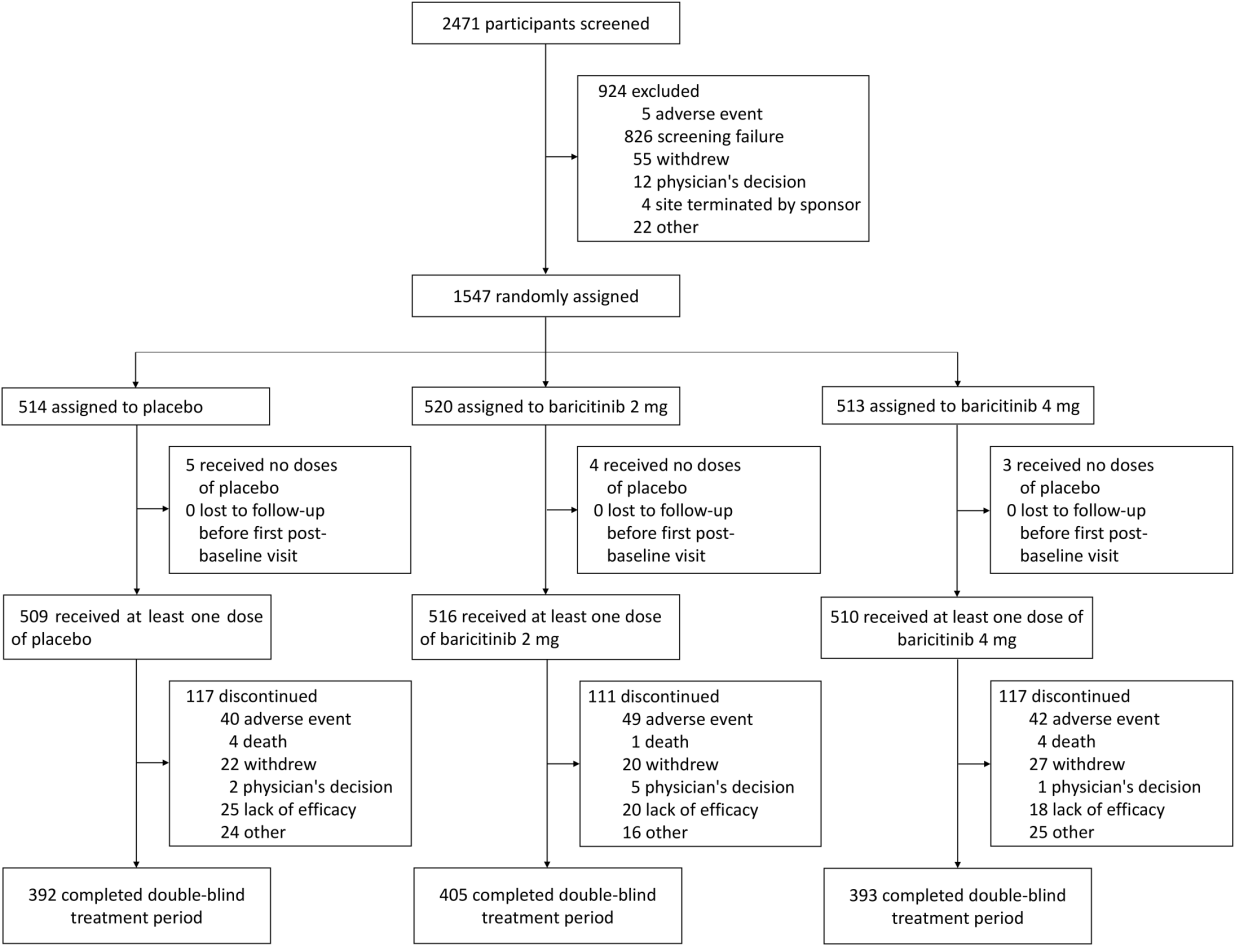
Patient inclusion flow chart.

Of the 1535 patients enrolled in SLE-BRAVE-I and II, 510, 516, and 509 patients were randomized to once-daily baricitinib 4 mg plus SOC, baricitinib 2 mg plus SOC, or placebo plus SOC at baseline, respectively. 1190 participants completed the 52-week study (Fig 1) and 345 dropped out of the study during this period. Twelve (0.8%) participants received no dose of any study treatment, so they were excluded from the analyses of efficacy and safety. Missing data (98 (19%) of participants from the baricitinib 4 mg group, 79 (15%) participants from the baricitinib 2 mg group, and 83 (16%) participants from the placebo group) were excluded.

Baseline demographics of each group are shown in Table 1. 1444 (94%) participants were female. At baseline, the mean age was 42.8 (standard deviation (SD) 12.7) years. The median duration was 6.80 (IQR 2.75–12.64) years and mean duration of SLE was 8.9 (7.9) years at baseline. Mean SLEDAI-2K score of participants was 10.1 (SD 3.1) at baseline. At baseline, the majority (1200 [78%]) of participants were receiving glucocorticoids with a mean prednisone dose (or equivalent) of 9.7 (SD 5.8) mg per day, 1243 (81%) were receiving anti-malarials, and 849 (55%) were receiving immuno-suppressants.

**Table 1:** Baseline demographics and clinical characteristics.

	Placebo (n = 509)	Baricitinib 2 mg (n = 516)	Baricitinib 4 mg (n = 510)
Mean age, years	42.7 (12.8)	42.8 (12.7)	41.9 (12.5)
Mean time since onset of SLE, years	9.2 (7.9)	8.9 (7.7)	8.6 (8.0)
Sex			
Female	478 (94%)	484 (94%)	482 (95%)
Male	31 (6%)	32 (6%)	28 (5%)
Race	502	508	502
America Indian or Alaska Native	26 (5%)	27 (5%)	20 (4%)
Asian	104 (20%)	105 (20%)	104 (20%)
Black or African American	53 (10%)	46 (9%)	56 (11%)
Native Hawaiian or other Pacific Islander	0	0	1 (0.2%)
White	313 (61%)	324 (63%)	317 (62%)
Multiple	6 (1%)	6 (1%)	4 (1%)
Ethnicity (USA only)	97	99	99
Hispanic or Latino	13 (13%)	24 (24%)	19 (19%)
Not Hispanic or Latino	82 (85%)	73 (74%)	78 (79%)
Not reported	2 (2%)	2 (2%)	2 (2%)
Region	509	516	510
North America	97 (19%)	99 (19%)	99 (19%)
Asia	62 (12%)	65 (13%)	62 (12%)
Europe	119 (23%)	121 (23%)	120 (24%)
Central America, South America, and Mexico	133 (26%)	131 (25%)	129 (25%)
Rest of world	98 (19%)	100 (19%)	100 (20%)
Disease characteristics			
Antinuclear antibodies titer ≥1:80	454 (93%) of 487	456 (91%) of 500	465 (94%) of 496
Mean anti-dsDNA, IU/mL	82.73 (324.62)	106.4 (509.17)	122.14 (578.4)
Anti-dsDNA ≥ 15 IU/mL	195 (38%)	220 (43%)	197 (39%)
Mean C3, g/L	1.06 (0.33)	1.06 (0.31)	1.07 (0.33)
C3 < 90.0 mg/dL	169 (33%)	164 (32%)	159 (31%)
Mean C4, g/L	0.19 (0.10)	0.18 (0.10)	0.19 (0.10)
C4 < 10.0 mg/dL	103 (20)	108 (21%)	110 (22%)
Mean urine protein:creatinine ratio, mg/mmol	23.46 (35.76)	23.71 (27.41)	22.57 (32.79)
Urine protein:creatinine ratio <50 mg/mmol	467 (92%)	462 (90%)	469 (92%)
Urine protein:creatinine ratio ≥50 mg/mmol	40 (8%)	54 (10%)	41 (8%)
Mean eGFR, mL per min per 1.73m²	94.7 (27.19)	94.02 (25.2)	98.15 (26.19)
Concomitant medications			
Glucocorticoids	402 (79%)	404 (78%)	394 (77%)
Mean prednisone dose (or equivalent), mg/day	9.3 (5.0)	10.0 (6.5)	9.9 (5.8)
Prednisone dose (or equivalent) ≥10 mg/day	213 (42%)	215 (42%)	209 (41%)
Antimalarials	422 (83%)	402 (78%)	419 (82%)
Immunosuppressants	290 (57%)	285 (55%)	274 (54%)
Methotrexate	109 (21%)	103 (20%)	112 (22%)
Azathioprine	85 (17%)	102 (20%)	75 (15%)
Mycophenolate mofetil	68 (13%)	64 (12%)	59 (12%)
Non-steroidal anti-inflammatory drug	114 (22%)	131 (25%)	138 (27%)
Mean SLEDAI-2K score	10.1 (3.1)	10.2 (3.2)	10 (3.0)
SLEDAI-2K score ≥10	291 (57%)	301 (58%)	290 (57%)
SLEDAI-2K organ system involvement			
CNS	0	0	0
Vascular	27 (5%)	26 (5%)	24 (5%)
Musculoskeleta	499 (98%)	503 (97%)	498 (98%)
Rena	33 (6%)	46 (9%)	39 (8%)
Mucocutaneous	487 (96%)	498 (97%)	492 (96%)
Cardiovascular and respiratory	14 (3%)	12 (2%)	17 (3%)
Immunological	272 (53%)	274 (53%)	269 (53%)
Constitutional	12 (2%)	13 (3%)	13 (3%)
Haematological	33 (6%)	32 (6%)	38 (7%)
≥1 BILAG A scores	326 (64%)	361 (70%)	326 (64%)
≥2 BILAG B scores	154 (30%)	133 (26%)	161 (32%)
Mean Physician’s Global Assessment score			
Mean CLASI activity score	6.6 (6.1)	6.6 (6.8)	6.4 (5.7)
Mean tender joint count	10.3 (7.1)	10.3 (6.7)	10.7 (7.1)
Mean swollen joint count	6.75 (5.5)	6.77 (5.1)	6.8 (5.2)
Mean SLICC/ACR Damage Index score	0.63 (1.1)	0.64 (1.1)	0.6 (1.0)

Data are mean (SD) or n (%); Asia; included China and Taiwan; Central America, South America, and Mexico included Brazil and Mexico; Europe included Austria, Belgium, Croatia, Czech Republic, Germany, Greece, Hungary, Switzerland, the Netherlands, and the UK; North America uncluded the USA; and rest of world included Australia, Israel, and Russia; BILAG, British Isles Lupus Assessment Group; CLASI, Cutaneous Lupus Erythematosus Disease Area and Severity Index; eGFR, estimated glomerular filtration rate; SLE, systemic lupus erythematosus; SLEDAI-2K, Systemic Lupus Erythematosus Disease Activity Index 2000; SLICC/ACR, Systemic International Collaborating Clinics/American College of Lupus Rheumatology.

**Fig 2:**
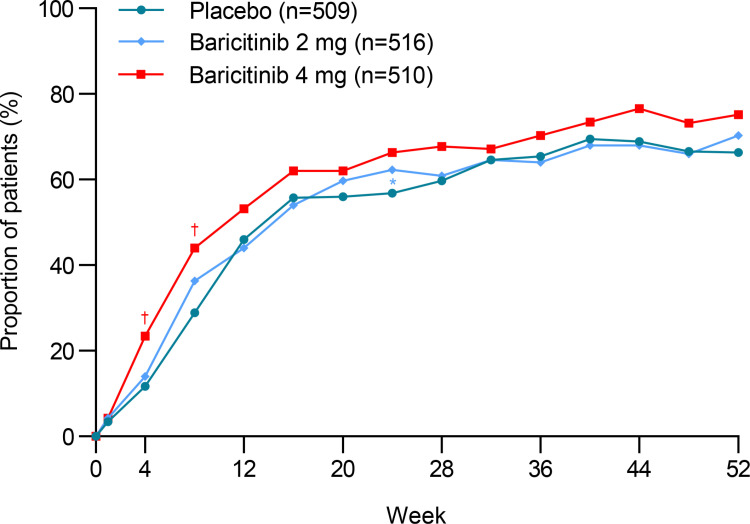
SRI-4 response through 52 weeks in overall study population. Proportion of patients reaching an SRI-4 response over the 52-week study period; SRI-4: Systemic Lupus Erythematosus Responder Index-4; *P ≤ 0.05; †P ≤ 0.01.

**Fig 3:**
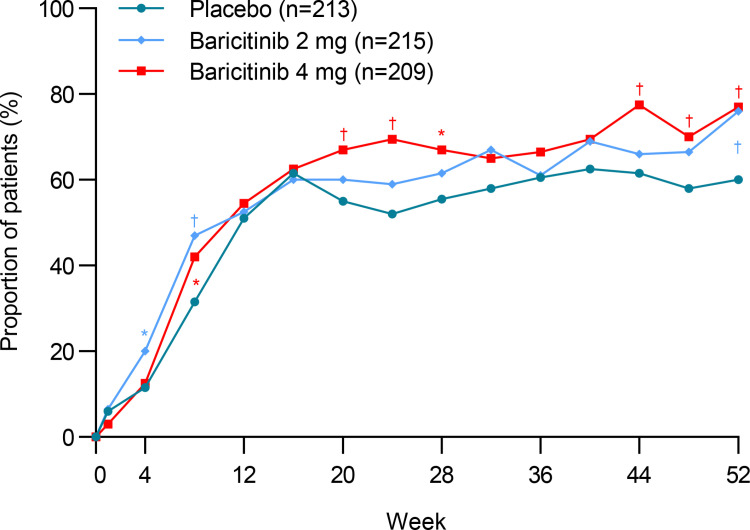
Proportion of patients achieving an SRI-4 response who had glucocorticoid **≥ 10mg/day at baseline.** I- SRI-4: Systemic Lupus Erythematosus Responder Index-4; *P ≤ 0.05; †P ≤ 0.01.

At week 52, there was no significant difference between participants given baricitinib 4 mg (263 [52%]; 1.27; 0.99, 1.63) or baricitinib 2 mg (246 [48%]; 1.09; 0.85, 1.39), and placebo (232 [46%]) in SRI-4 response (Table 2). SRI-4 responses at all assessed timepoints are shown in Fig 2. However, in the subgroup of participants who had glucocorticoid ≥ 10mg/day at baseline, significant difference was found in the SRI-4 response between participants receiving baricitinib 4 mg (154 [74%]; 2.32; 1.50, 3.61) or baricitinib 2 mg (152 [71%]; 1.90; 1.23, 2.95) and placebo, at week 52 (Fig 3). There was significant difference between participants

**Table 2:** Primary and key secondary outcomes in the intention-to-treat population at week 52.

	Placebo (n = 509)	Baricitinib 2 mg (n = 516)	Baricitinib 2 mg odds ratio (95% CI); difference with placebo (95% CI); p value	Baricitinib 4 mg (n = 510)	Baricitinib 4 mg odds ratio (95% CI); difference with placebo (95% CI); p value
**Primary outcome**					
SRI-4^*^^,^^†^^,^^‡^	232 (46%)	246 (48%)	1.09 (0.85, 1.39); 0.50	263 (52%)	1.27 (0.99, 1.63); 0.56
Reduction of ≥ 4 points from baseline in SLEDAI-2K score^*^^,^^†^^,^^‡^	233 (46%)	252 (49%)	1.13 (0.88, 1.45); 0.33	269 (53%)	1.32 (1.03, 1.69); 0.26
No new BILAG A and no more than one new BILAG B disease activity score^*^^,^^†^^,^^‡^	378 (74%)	391 (76%)	1.08 (0.82, 1.44); 0.58	388 (76%)	1.10 (0.83, 1.46); 0.50
No worsening (defined as an increase of ≥ 0.3 points [10 mm] from baseline) in the PGA^*^^,^^†^	381 (75%)	394 (76%)	1.08 (0.82, 1.44); 0.58	388 (76%)	1.07 (0.80, 1.42); 0.65
**Major secondary outcomes n (%)**					
SRI-4 (week 24)^*^^,^^†^^,^^‡^	197 (39%)	218 (42%)	1.16 (0.90, 1.49); 0.25	225 (44%)	1.25 (0.97, 1.61); 0.79
Participants with ≥1 severe flare^‡^	64 (13%)	63 (12%)	0.97 (0.67, 1.40); 0.86	55 (11%)	0.84 (0.57, 1.23); 0.37
Glucocorticoid sparing^*^^,^^†^^,^^§^	69/221 (31%)	65/220 (30%)	0.87 (0.55, 1.36); 0.70	72/211 (34%)	1.29 (0.82, 2.05); 0.52
LLDAS^*^^,^^†^^,^^‡^	125 (25%)	127 (25%)	1.00 (0.75, 1.33); 0.98	139 (27%)	1.15 (0.87, 1.52); 0.33
**Other secondary outcomes**					
BICLA	216 (42%)	228 (44%)	1.07 (0.84, 1.37); 0.57	240 (47%)	1.21 (0.94, 1.54); 0.14
SLEDAI-2K remission of arthritis or rash	251 (49%)	257 (50%)	1.02 (0.80, 1.30); 0.87	280 (55%)	1.25 (0.98, 1.60); 0.74
≥50% reduction in CLASI activity score^*^^,^^†^^,^**	63/108 (58%)	54/97 (56%)	0.96 (0.75, 1.22); 0.70	53/93 (57%)	0.95 (0.54, 1.66); 0.85

Data are n (%), n/N (%), or least squares mean (SE), unless stated otherwise; Nominal, non-multiplicity-controlled p values are shown for all outcomes; BILAG: British Isles Lupus Assessment Group; CLASI: Cutaneous Lupus Erythematosus Disease Area and Severity Index; FACIT-Fatigue: Functional Assessment of Chronic Illness Therapy-Fatigue Scale; LLDAS, lupus low disease activity state; NA, not available; NRS: Numeric Rating Scale; PGA, Physician Global Assessment; SLE, Systemic Lupus Erythematosus; SLEDAI-2K: Systemic Lupus Erythematosus Disease Activity Index 2000; SRI: Systemic Lupus Erythematosus Responder Index;

*The number of patients is not the observed value but is estimated from the responder proportion of the multiple imputation results multiplied by the number of patients in the modified intention-to-treat population;

†Comparison is odds ratio;

‡Comparison is hazard ratio;

§Proportion of patients receiving >7·5 mg prednisone (or equivalent) at baseline able, decrease dose by ≥ 25%, a prednisone equivalent dose of ≤ 7·5 mg/day maintained between week 40 and week 52;

¶Comparison is least squares mean difference; ||Patients with CLASI,tal activity score ≥10 at baseline who achieved ≥50% reduction; ^**^Comparison is difference vs placebo.

**Fig 4:**
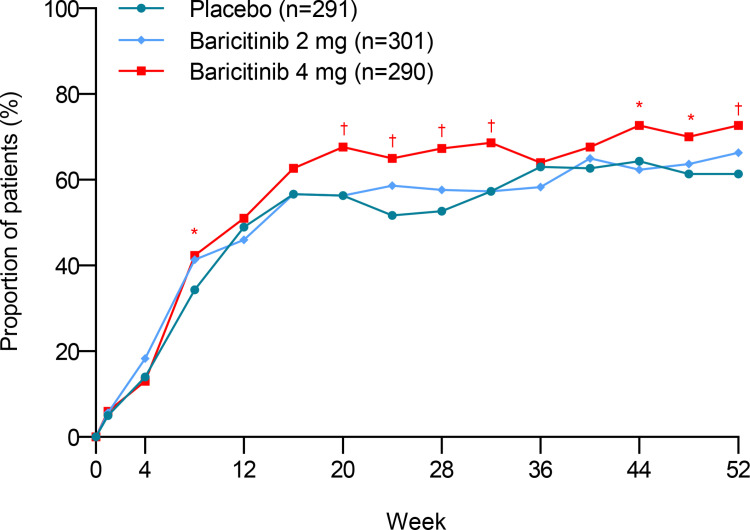
Proportion of patients achieving an SRI-4 response who had SLEDAI-2K **≥ 10 at baseline.** SLEDAI-2K: Systemic Lupus Erythematosus Disease Activity Index-2000; SRI-4: Systemic Lupus Erythematosus Responder Index-4; *P ≤ 0.05; †P ≤ 0.01.

treated with baricitinib 4 mg and placebo at week 52 (1.83; 1.27, 2.64; P < 0.01) in the subgroup of participants with a SLEDAI-2K score of 10 or more at baseline (Fig 4). For this subpopulation, at multiple timepoints besides week 52, the proportion of participants receiving baricitinib 4 mg achieved SRI-4 response was higher than that treated with placebo.

Given the fact that the primary endpoint has not been reached, major secondary outcomes were not considered to be of multiplicity-controlled significance, whatever their nominal *p* value were. No significant difference was found between baricitinib 4 mg or 2 mg and placebo in any non-multiplicity-controlled secondary outcomes or exploratory outcomes. For the subpopulation who were able to reduce the dose of prednisone or equivalent to 7.5 mg or less per day before week 52, the proportion of participants in the baricitinib 4 mg group was 34% (n = 72) (1.06; 0.71, 1.59) and in the baricitinib 2 mg group was 30% (n = 65) (0.92; 0.62, 1.39) compared with 31% (n = 69) in the placebo group. Significant difference was observed in median time to first severe flare between participants treated with baricitinib 4 mg group and placebo (2.25; 1.38, 3.64), but no significant difference between participants treated with baricitinib 2 mg and placebo (1.11; 0.64, 1.91). At week 52, the proportions of participants with LLDAS responses were 27% (n = 139) in the baricitinib 4 mg group (1.15; 0.87, 1.52) and 25% (n = 127) in the baricitinib 2 mg group (1.00; 0.75, 1.33), compared with 25% (n = 125) in the placebo group. Compared with placebo, baricitinib 4 mg or baricitinib 2 mg showed significant difference in changes from baseline in Worst Pain NRS and FACIT-Fatigue total score at week 52 (Table 2). Other secondary outcomes are shown in Table 2.

**Fig 5:**
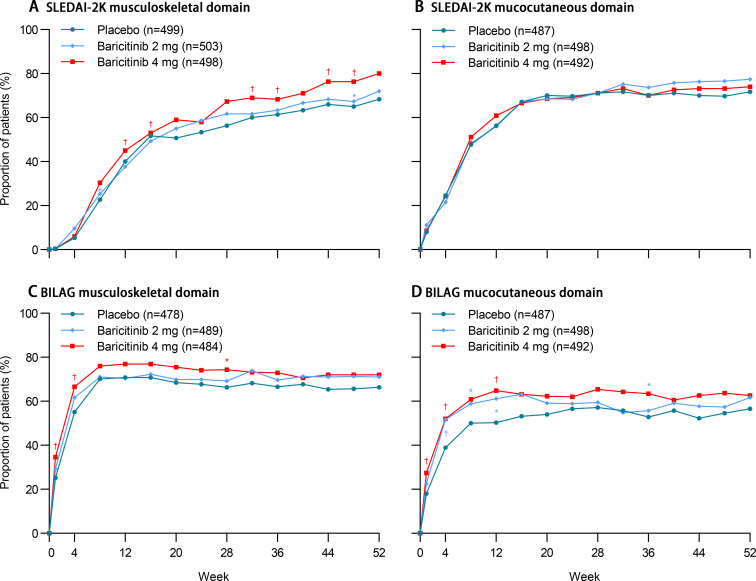
Improvement from baseline in SLEDAI-2K and BILAG organ systems through 52 weeks. (A) Im(A) Improvement from baseline in the SLEDAI-2K organ system for the musculoskeletal domain. (B) Improvement from baseline in the SLEDAI-2K organ system for the mucocutaneous domain. (C) Improvement from baseline in the BILAG organ system for the musculoskeletal domain. (D) Improvement from baseline in the BILAG organ system for the mucocutaneous domain.

At week 52, in non-multiplicity-controlled analyses of secondary outcomes, no improvements in any organ domains of interest were observed. However, improvements in the musculoskeletal SLEDAI-2K domain were seen in both the baricitinib 4 mg and baricitinib 2 mg groups at multiple timepoints (Fig 5A). No significant difference in the mucocutaneous SLEDAI-2K domain were seen in baricitinib 4 mg or baricitinib 2 mg group at any timepoint (Fig 5B). As a post-hoc analysis, the improvements in the musculoskeletal and mucocutaneous domains were also evaluated for the BILAG organ system. Improvements in the musculoskeletal domains were observed in the baricitinib 4 mg treatment group at multiple timepoints; and improvements in the mucocutaneous domains were also observed both in the baricitinib 4 mg and baricitinib 2 mg groups at multiple timepoints (Fig 5C and 5D).

The analysis population included participants who had a positive organ domain score in each assessment at baseline; BILAG: British Isles Lupus Assessment Group; SLEDAI-2K: Systemic Lupus Erythematosus Disease Activity Index-2000; *P ≤ 0.05; †Nominal P ≤ 0.01.

At least one treatment-emergent adverse event (AE) was observed for 408 (80%) participants in the baricitinib 4 mg group, 409 (79%) in the baricitinib 2 mg group, and 408 (80%) in the placebo group (Table 3). In most AEs, the severity is mild or moderate. Serious adverse events (SAEs) were reported in 55 (11%) participants in the baricitinib 4 mg group, 59 (11%) in the baricitinib 2 mg group, and 40 (8%) in the placebo group (Table 3). Four (1%) deaths were reported in participants randomized to baricitinib 4 mg group. Of these four deaths, 2 were due to myocardial infarctions, 1 was sepsis, and 1 was confirmed COVID-19-related acute respiratory failure (this one was randomly assigned to baricitinib 4 mg but received actually baricitinib 2 mg). One (0.2%) death was reported in participants assigned to baricitinib 2 mg. Four (1%) deaths were reported in the placebo group, all of whom were due to respiratory failure (three was confirmed COVID-19-related respiratory failure) (Table 3). Table 3 summarizes the withdrawal of treatment due to AEs. Compared with placebo (11 [2%] participants), more serious infections were reported in participants treated with baricitinib 4 mg (22 [4%] participants) and baricitinib 2 mg (20 [4%] participants) (Table 3). One (0.2%) adjudicated venous thrombotic event was reported in participants treated with baricitinib 4 mg, 3 (1%) in participants treated with baricitinib 2 mg, and 6 (1%) in the placebo group. Three (1%) adjudicated major cardiovascular events were reported in participants treated with baricitinib 4 mg, 1 (0.2%) in participants treated with baricitinib 2 mg, and none in participants treated with placebo (Table 3). There were 4 (1%) malignancies reported in participants treated with placebo, 3 (1%) in participants treated with baricitinib 2 mg, and 2 (0.4%) in participants treated with baricitinib 4 mg (Table 3). Laboratory results from patients would indicate no potential cases of Hy’s Law, which is defined as having a maximum post-baseline total bilirubin of twice or more upper limit of normal and a maximum post-baseline alanine aminotransferase or aspartate aminotransferase of concentrations triple or more upper limit of normal. Safety data are further described in Table 3 and S2-4 Tables.

**Table 3:** Overview of adverse events.

	Placebo (n = 509)	Baricitinib 2 mg (n = 516)	Baricitinib 4 mg (n = 510)
Treatment-emergent AEs	408 (80%)	409 (79%)	408 (80%)
Mild	174 (34%)	170 (33%)	171 (34%)
Moderate	199 (39%)	192 (37%)	189 (37%)
Severe	35 (7%)	47 (9%)	48 (9%)
SAEs	40 (8%)	59 (11%)	55 (11%)
Death	4 (1%)	1 (0.2%)	4 (1%)
Discontinuation from study because of an AE	44 (8.6%)	48 (9%)	46 (9%)
Infections	260 (51%)	266 (52%)	264 (52%)
Serious infections	11 (2%)	20 (4%)	22 (4%)
Herpes simplex	16 (3%)	19 (4%)	11 (2%)
Herpes zoster virus infection	17 (3%)	17 (3%)	28 (5%)
Opportunistic infections	23 (5%)	30 (6%)	38 (7%)
Major adverse cardiovascular events^*^	0	1 (0.2%)	3 (1%)
Cardiovascular death	0	0	2 (0.4%)
Myocardial infarction	0	1 (0.2%)	1 (0.2%)
Stroke	0	0	1 (0.2%)
Arterial thromboembolic events^*^	0	0	0
Venous thromboembolic events^*^	6 (1%)	3 (1%)	1 (0.2%)
Deep-vein thrombosis	1 (0.2%)	3 (1%)	0
Pulmonary embolism	1 (0.2%)	2 (0.4%)	0
Other^†^	4 (1%)	0	1 (0.2%)
Malignancies	4 (1%)	3 (1%)	2 (0.4%)
Non-melanoma skin cancer	2 (0.4%)	0	0
Malignancies other than non-melanoma skin cancer	2 (0.4%)	3 (1%)	2 (0.4%)
Gastrointestinal perforations	0	1 (0.2%)	0
Hepatic disorders	21 (4%)	38 (8%)	22 (4%)

Data are n (%); AE, adverse event; SAE, Serious adverse events; AEs that occurred between baseline and week 52 and up to 28 days after treatment are shown;

*Positively adjudicated by an independent external masked clinical event committee;

†Other events were all classified as non-superficial below-knee thrombosis.

## Discussion

With various and selective inhibitory profiles, JAK inhibitors have enabled a shift in the treatment of multiple autoimmune diseases [26–29]. The etiology of SLE, RA, AA, and other inflammatory diseases involves the intracellular signaling of multiple cytokines, JAK inhibitors can block these signal pathways [30,31].

Baricitinib is an oral small-molecule inhibitor of JAK1/2 signaling which is approved for the management of RA, juvenile idiopathic arthritis (JIA), atopic dermatitis (AD), AA, and coronavirus disease 2019 (COVID-19). Substantial evidence backs the role of pathways blocked by baricitinib in the pathogenesis of systemic lupus erythematosus [32], including evidence showed effects on expression of serum cytokines, IFNs responsive genes, and JAK-STAT pathway genes [13,18–20], and positive results of a phase Ⅲ study (SLE-BRAVE-I), which showed that baricitinib 4 mg plus SOC was superior to placebo in conjunction with SOC in reaching SRI-4 response at week 52 [21]. However, the result was not replicated in another phase Ⅲ study (SLE-BRAVE-II) [22], necessitating a pooled analysis of the only two available phase Ⅲ randomized clinical trials to investigate the efficacy and safety of baricitinib 4 mg and baricitinib 2 mg versus placebo in active SLE participants treated with SOC.

In this study, the proportion of participants reaching an SRI-4 response was not achieved with once-daily oral baricitinib 4 mg or baricitinib 2 mg plus SOC compared with placebo. The reasons for not achieving SRI-4 response at week 52 are detailed in S5 Table. In any non-multiplicity-controlled secondary outcomes or exploratory outcomes, no difference was found between baricitinib 4 mg or 2 mg and placebo. However, in some clinically important subpopulations, SRI-4 response was achieved, such as in participants treated with a glucocorticoid dose at baseline of 10 mg per day or higher of prednisone or equivalent (baricitinib 4 mg or baricitinib 2 mg) and in participants with highly active disease (SLEDAI-2K score at baseline >10) (baricitinib 4 mg). These findings confirm the efficacy of baricitinib for treating SLE in these subgroups. Patient selection might not be a major factor affecting the efficacy [8], because the patients profile in this study, including concomitant medication and organ involvement, was similar to those of other recently published SLE clinical trials [21,22,8]. Patients with baseline glucocorticoid use of ≥ 10 mg/day often exhibit more severe disease activity or flares that necessitate aggressive treatment. Baricitinib functions by inhibiting JAK enzymes, which can help reduce inflammation and immune system activity in patients with SLE. Clinical trials suggest that baricitinib may improve disease activity in SLE patients, particularly those who have inadequate responses to conventional therapies, including glucocorticoids [15]. Identifying biomarkers that predict response to treatment in SLE is essential for personalizing therapy and improving patient outcomes. Key biomarkers include anti-dsDNA antibodies, complement levels (C3 and C4), and SLEDAI scores, among others.

In general, baricitinib 4 mg or baricitinib 2 mg for treating SLE did not increase the incidence of treatment-emergent adverse events (TEAEs). There were no new safety signals observed, and safety profile of baricitinib was consistent with what is known. Patients receiving baricitinib 4 mg had higher rates of herpes zoster infections, which is consistent with other treatment groups and is a typical complication of JAK1 inhibitors [15]. In both the baricitinib 4 mg and baricitinib 2 mg groups, the serious infection was higher, but within the expected range. Other TEAEs and SAEs, including MACE, infections, malignancy, and hepatic disorders, were all within the expected range. It is important to note that in the population at high risk of VTEs, there was no gastrointestinal perforations, or VTE excess [33].

This study also has several limitations. Firstly, similar to other trials in SLE, this study did not include patients with central nervous system (CNS) and severe active renal disease. Secondly, the overall public health context during COVID-19 created an acute focus on health issues, potentially fostering a placebo-enhancing environment in which patients were more inclined to attribute improvements or changes in symptoms to their treatment, even when they received a placebo. Part of the two trials took place during the COVID-19 pandemic, and the placebo rates were higher compared with other recent SLE studies [34,35], which may cover up the efficacy of baricitinib for treating SLE to some extent. Thirdly, steroid tapering was not carried out as rigorously as it might have been in other recent trials, which could lead to an increase in placebo response rate. Lastly, analysis should be interpreted with caution due to missing data and inadequate analytical power in the subgroups of participants who received glucocorticoid dose at baseline of 10 mg daily or higher of prednisone or equivalent or SLEDAI-2K score of 10 or higher.

## Conclusions

In conclusion, this pooled analysis of the SLE-BRAVE-I and Ⅱ trials demonstrated that once-daily oral baricitinib 4 mg or baricitinib 2 mg plus SOC did not reduce the overall disease activity. However, baricitinib is likely to be a good treatment option for SLE in certain subpopulation, such as in participants treated with a glucocorticoid dose at baseline of 10 mg daily or higher of prednisone or equivalent (baricitinib 4 mg and baricitinib 2 mg) and in participants with highly active disease (SLEDAI-2K score at baseline >10) (baricitinib 4 mg). Furthermore, baricitinib 4 mg and baricitinib 2 mg for treating SLE did not increase the incidence of TEAEs. These data are invaluable for healthcare professionals to provide consultation on treatment expectations and potential benefits to patients.

## Supporting information

S1 Fig Study Design. (TIF)

S1 Table Description of Efficacy Measures. (DOCX)

S2 Table Treatment Emergent Adverse Events Occurring in ≥ 5% of Patients in Either Baricitinib Dose Group, Weeks 0–52 and up to 28 Days Post-Treatment. (DOCX)

S3 Table Serious Adverse Events by System Organ Class, Weeks 0–52 and up to 28 Days Post-Treatment. (DOCX)

S4 Table Serious Infections and Infestations, Weeks 0–52 and up to 28 Days Post-Treatment. (DOCX)

S5 Table Reasons for not Achieving SLE Responder Index-4 at Week 52. (DOCX)

## Abbreviations

AE: Adverse event
AA: Alopecia areata
AD: Atopic dermatitis
CNS: Central nervous system
CONSORT: Consolidated Standards of Reporting Trial
COVID-19: Coronavirus disease 2019
FACIT: Functional Assessment of Chronic Illness Therapy
IFN: Interferons
IL: Interleukin
JAK: Janus kinases
JIA: Juvenile idiopathic arthritis
LLDAS: Lupus low disease activity state
MACE: Major adverse cardiovascular events
NRS: Numeric Rating Scale
OR: Odds ratio
RA: Rheumatoid arthritis
SAE: Serious adverse events
SRI: SLE Responder Index
SLE: Systemic lupus erythematosus
SOC: Standard of care
TEAE: Treatment-emergent adverse event
TNF: Tumor necrosis factor
VTE: Venous thromboembolic event
