# Supplementary figures and images for: Clinical outcomes of baricitinib in patients with systemic lupus erythematosus: Pooled analysis of SLE-BRAVE-I and SLE-BRAVE-II trials
Source: PLoS One. 2025 Apr 30;20(4):e0320179. doi: 10.1371/journal.pone.0320179 (PMC12043178; doi:10.1371/journal.pone.0320179)

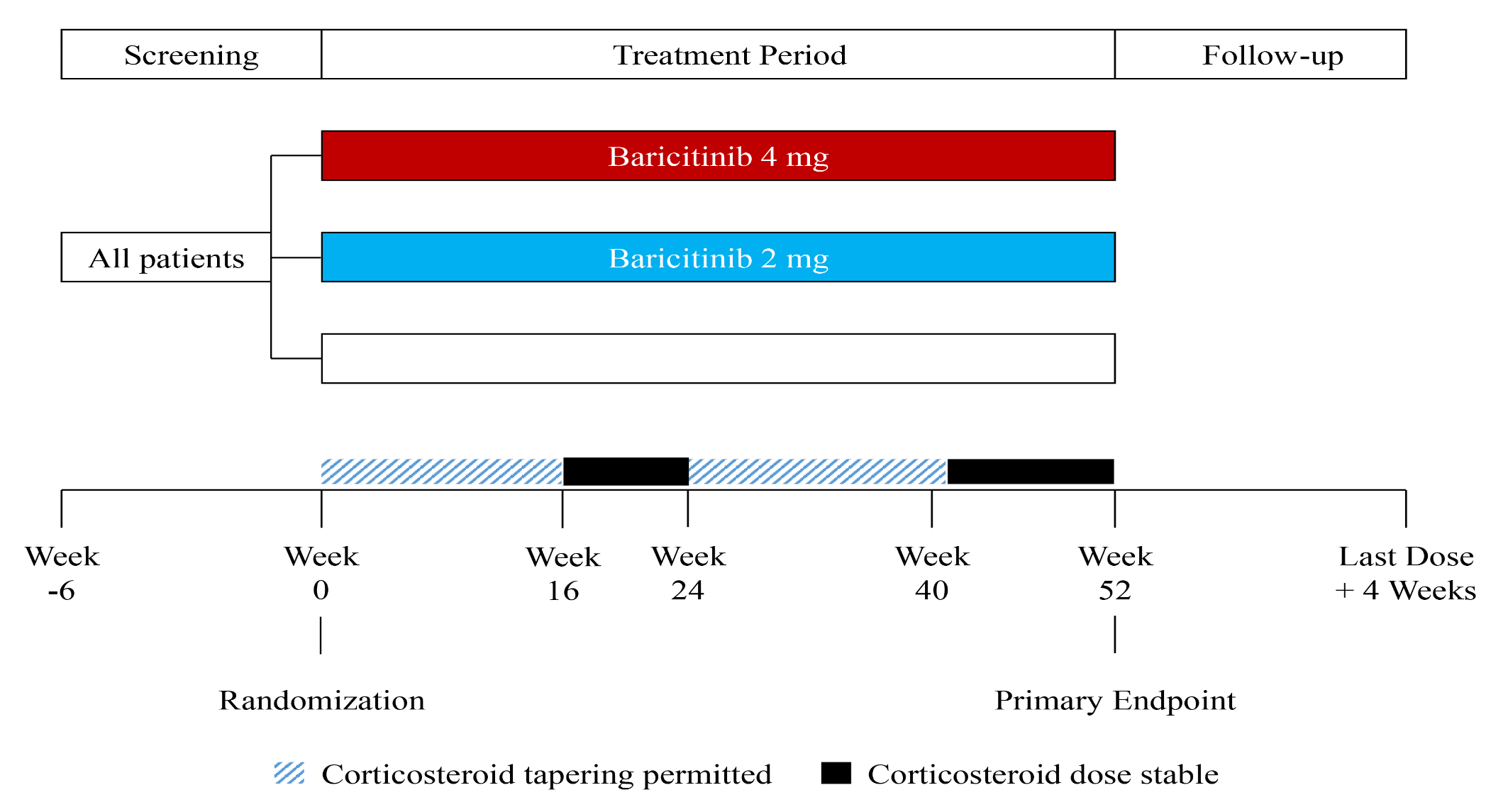

Supplement: S1 Fig — (TIF) [file pone.0320179.s001.tif]
