# Supplementary material for: Clinical outcomes of baricitinib in patients with systemic lupus erythematosus: Pooled analysis of SLE-BRAVE-I and SLE-BRAVE-II trials
Source: PLoS One. 2025 Apr 30;20(4):e0320179. doi: 10.1371/journal.pone.0320179 (PMC12043178; doi:10.1371/journal.pone.0320179)
Supplement: S1 Table — (DOCX) [file pone.0320179.s002.docx]

| **S1 Table. Description of Efficacy Measures.** | | | | |
| --- | --- | --- | --- | --- |
| **Measure** | **Definition** | **Scale** | **Reference** |  |
| Systemic Lupus Erythematosus Responder Index-4 (SRI-4) | Composite responder index based on improvement in disease activity (at least four point improvement in SLEDAI score) without worsening of the overall condition (no worsening in PGA) or the development of significant disease activity in new organ systems (no new BILAG A or >1 new BILAG B). | N/A | 1 |  |
| Systemic Lupus Erythematosus Disease Activity Index 2000 (SLEDAI-2K) | Global SLE disease activity index that focuses on high-impact disease manifestations across 9 organ systems. It includes 24 clinical and laboratory variables with manifestations weighted by the affected organ system. | Scores range from 0 to 105, with higher scores indicating more severe disease | 2 |  |
| British Isles Lupus Assessment Group BILAG 2004 Index | Global SLE disease activity index designed on the basis of the physician’s intention to treat, focusing on changes in disease manifestations. The instrument assesses 97 clinical signs, symptoms, and laboratory parameters across 9 organ systems. | Letter score assigned to each organ system with following indications: A=severe, B=moderate, C=mild, D=inactive with prior history, and E=inactive with no history | 3 |  |
| Physician’s Global Assessment of Disease Activity | Physician’s Global Assessment of Disease Activity The physician’s assessment of the patient’s overall disease activity due to SLE, as compared with all possible patients with SLE. | Visual-analog scale ranging from 0 (0 mm) to 3 (100 mm), with higher values indicating, more severe disease | 4 |  |
| SELENA-SLEDAI Flare Index (SSFI) | An index defining SLE flares using the SLEDAI-2K score, disease activity scenarios, treatment changes, and Physician’s Global Assessment of Disease Activity. | Mild/moderate or severe flare | 5 |  |
| Lupus Low Disease Activity State (LLDAS) | A state of low disease activity based on SLEDAI score, absence of SLE disease activity in major organ systems and new disease activity, Physician’s Global Assessment, and concomitant medication usage. | N/A | 6 |  |
| Cutaneous Lupus Erythematosus Disease Area and Severity Index (CLASI) | Index used to assess cutaneous manifestations of SLE summarizing the activity of the disease. | Scores range from 0 to 70, with higher scores indicating more severity | 7 |  |
| Systemic Lupus Erythematosus International Collaborating Clinics/ American College of Rheumatology (SLICC/ACR) Damage Index | Index recording damage occurring in patients with SLE regardless of its cause including specific comorbidities associated with SLE that may be due to treatment-related toxicity. The index is scored on 41 items in 12 organ systems. | Scores range from 0 to 45, with higher scores indicating more damage |  |  |
| Tender/Swollen Joint Count (28 Joints) | 28 joints, 14 on each side of the patient’s body, examined and assessed as tender or not tender (for tender joint count) and as swollen or not swollen (for swollen joint count): the two shoulders, the two elbows, the two wrists, the ten metacarpophalangeal joints, the two interphalangeal joints of the thumb, the eight proximal interphalangeal joints, and the 2 knees. |  |  |  |
| Worst Joint Pain NRS | A value recorded by patients based on the worst level of joint pain experienced in the last 24 hours. | Numeric rating scale ranging from 0 to 10 with 0=no joint pain, 10=joint pain as bad as you can imagine |  |  |
| Worst Pain NRS | A value recorded by patients based on the worst level of pain experienced over the last24 hours. | Numeric rating scale ranging from 0 to 10 with 0=no pain, 10=pain as bad as you can imagine |  |  |
| Worst Fatigue NRS | A value recorded by patients based on the worst level of fatigue experienced over the last 24 hours. | Numeric rating scale ranging from 0 to 10 with 0=no fatigue, 10=fatigue as bad as you can imagine |  |  |
| Functional Assessment of Chronic Illness Therapy (FACIT)–Fatigue Scale | A brief, 13-item, symptom-specific questionnaire that specifically assesses the self-reported severity of fatigue and its impact upon daily activities and functioning. The FACIT-Fatigue uses 0 (“not at all”) to 4 (“very much”) NRS to assess fatigue and its impact in the past 7 days. | Scores range from 0 to 52 with higher scores indicating less fatigue |  |  |
| **References**  1. Furie RA, Petri MA, Wallace DJ, et al. Novel evidence-based systemic lupus erythematosus responder index. Arthritis Rheum 2009; 61: 1143-51.  2. Gladman DD, Ibanez D, Urowitz MB. Systemic lupus erythematosus disease activity index 2000. J Rheumatol 2002; 29: 288-91.  3. Yee CS, Farewell V, Isenberg DA, et al. British Isles Lupus Assessment Group 2004 index is valid for assessment of disease activity in systemic lupus erythematosus. Arthritis Rheum 2007; 56: 4113-9.  4. Petri M, Buyon J, Kim M. Classification and definition of major flares in SLE clinical trials. Lupus 1999; 8: 685-91.  5. Golder, V., et al., Lupus low disease activity state as a treatment endpoint for systemic lupus erythematosus: a prospective validation study. The Lancet Rheumatology, 2019; 1(2): p. e95-e102.  6. Albrecht J, Taylor L, Berlin JA, et al. The CLASI (Cutaneous Lupus Erythematosus Disease Area and Severity Index): an outcome instrument for cutaneous lupus erythematosus. J Invest Dermatol 2005; 125: 889-94.  7. Gladman D, Ginzler E, Goldsmith C, et al. The development and initial validation of the Systemic Lupus International Collaborating Clinics/American College of Rheumatology damage index for systemic lupus erythematosus. Arthritis Rheum 1996; 39: 363-9 | | | | |
