# Supplementary material for: Clinical outcomes of baricitinib in patients with systemic lupus erythematosus: Pooled analysis of SLE-BRAVE-I and SLE-BRAVE-II trials
Source: PLoS One. 2025 Apr 30;20(4):e0320179. doi: 10.1371/journal.pone.0320179 (PMC12043178; doi:10.1371/journal.pone.0320179)
Supplement: S2 Table — (DOCX) [file pone.0320179.s003.docx]

| **S2 Table. Treatment Emergent Adverse Events Occurring in ≥5% of Patients in Either Baricitinib Dose Group, Weeks 0-52 and up to 28 Days Post-Treatment.** | | | | |
| --- | --- | --- | --- | --- |
| **MedDRA Preferred Term, n (%)** | **Placebo (N=509)** | **Baricitinib 2 mg (N=516)** | **Baricitinib 4 mg (N=510)** |  |
| Urinary tract infection | 51 (10.0) | 61 (11.8) | 59 (11.6) |  |
| Headache | 49 (9.6) | 41 (7.9) | 39 (7.7) |  |
| Upper respiratory tract infection | 30 (5.9) | 38 (7.4) | 38 (7.5) |  |
| Nasopharyngitis | 40 (7.9) | 38 (7.4) | 39 (7.7) |  |
| Hypertension | 19 (3.7) | 29 (5.6) | 30 (5.9) |  |
| Neutropenia | 18 (3.5) | 14 (2.7) | 26 (5.1) |  |
| MedDRA, Medical Dictionary for Regulatory Activities; N, number of patients in the analysis; n, number of patients in the specified category. | | | | |
