# Supplementary material for: Clinical outcomes of baricitinib in patients with systemic lupus erythematosus: Pooled analysis of SLE-BRAVE-I and SLE-BRAVE-II trials
Source: PLoS One. 2025 Apr 30;20(4):e0320179. doi: 10.1371/journal.pone.0320179 (PMC12043178; doi:10.1371/journal.pone.0320179)
Supplement: S3 Table — (DOCX) [file pone.0320179.s004.docx]

| **S3 Table. Serious Adverse Events by System Organ Class, Weeks 0-52 and up to 28 Days Post-Treatment.** | | | |  |
| --- | --- | --- | --- | --- |
| **MedDRA Preferred Term, n (%)** | **Placebo (N=509)** | **Baricitinib 2 mg (N=516)** | **Baricitinib 4 mg (N=510)** |  |
| Infections and infestations | 11 (2.2) | 20 (3.9) | 22 (4.3) |  |
| Musculoskeletal and connective tissue disorders | 5 (1.0) | 9 (1.7) | 8 (1.6) |  |
| Injury, poisoning and procedural complications | 5 (1.0) | 3 (0.6) | 5 (1.0) |  |
| Gastrointestinal disorders | 4 (0.8) | 9 (1.7) | 4 (0.8) |  |
| Neoplasms benign, malignant and unspecified (incl cysts and polyps) | 4 (0.8) | 3 (0.6) | 3 (0.6) |  |
| Respiratory, thoracic and mediastinal disorders | 4 (0.8) | 3 (0.6) | 0 |  |
| Vascular disorders | 3 (0.6) | 2 (0.4) | 0 |  |
| Renal and urinary disorders | 2 (0.4) | 4 (0.8) | 3 (0.6) |  |
| Nervous system disorders | 2 (0.4) | 2 (0.4) | 3 (0.6) |  |
| General disorders and administration site conditions | 2 (0.4) | 1 (0.2) | 3 (0.6) |  |
| Metabolism and nutrition disorders | 2 (0.4) | 1 (0.2) | 0 |  |
| Reproductive system and breast disorders | 1 (0.2) | 3 (0.6) | 3 (0.6) |  |
| Cardiac disorders | 1 (0.2) | 2 (0.4) | 6 (1.2) |  |
| Hepatobiliary disorders | 1 (0.2) | 2 (0.4) | 1 (0.2) |  |
| Blood and lymphatic system disorders | 0 | 2 (0.4) | 3 (0.6) |  |
| Pregnancy, puerperium and perinatal conditions | 0 | 1 (0.2) | 2 (0.4) |  |
| MedDRA, Medical Dictionary for Regulatory Activities; N, number of patients in the analysis; n, number of patients in the specified category. | | | | |
