# Supplementary material for: Clinical outcomes of baricitinib in patients with systemic lupus erythematosus: Pooled analysis of SLE-BRAVE-I and SLE-BRAVE-II trials
Source: PLoS One. 2025 Apr 30;20(4):e0320179. doi: 10.1371/journal.pone.0320179 (PMC12043178; doi:10.1371/journal.pone.0320179)
Supplement: S4 Table — (DOCX) [file pone.0320179.s005.docx]

| **S4 Table. Serious Infections and Infestations, Weeks 0-52 and up to 28 Days Post-Treatment.** | | | |
| --- | --- | --- | --- |
| **MedDRA Preferred Term, n (%)** | **Placebo (N=509)** | **Baricitinib 2 mg (N=516)** | **Baricitinib 4 mg (N=510)** |
| **Infections and infestations** | 11 (2.2) | 20 (3.9) | 22 (4.3) |
| Pneumonia | 3 (0.3) | 5 (1.0) | 4 0.8) |
| Pneumonia bacterial | 2 (0.4) | 0 | 2 (0.4) |
| Urinary tract infection | 2 (0.4) | 0 | 1 (0.2) |
| COVID-19 | 1 (0.2) | 3 (0.6) | 2 (0.4) |
| COVID-19 pneumonia | 1 (0.2) | 2 (0.4) | 1 (0.2) |
| Gastroenteritis | 1 (1.2) | 1 (0.2) | 0 |
| Herpes zoster meningomyelitis | 1 (0.2) | 0 | 2 (0.4) |
| Appendicitis | 0 | 0 | 2 (0.4) |
| Infection | 0 | 0 | 4 0.8) |
| Tubo-ovarian abscess^*^a | 0 | 2 (0.4) | 0 |
| Bronchitis | 0 | 2 (0.4) | 1 (0.2) |
| MedDRA, Medical Dictionary for Regulatory Activities; N, number of patients in the analysis; n, number of patients in the specified category.  ^*^Denominator adjusted because gender-specific event for females N=478 (placebo) N=484 (baricitinib 2 mg) N=482 (baricitinib 4 mg). | | | |
