# Supplementary material for: Clinical outcomes of baricitinib in patients with systemic lupus erythematosus: Pooled analysis of SLE-BRAVE-I and SLE-BRAVE-II trials
Source: PLoS One. 2025 Apr 30;20(4):e0320179. doi: 10.1371/journal.pone.0320179 (PMC12043178; doi:10.1371/journal.pone.0320179)
Supplement: S5 Table — (DOCX) [file pone.0320179.s006.docx]

| **S5 Table. Reasons for not Achieving SLE Responder Index-4 at Week 52. S5 Table** | | | |
| --- | --- | --- | --- |
|  | **Placebo (N=509)** | **Baricitinib 2 mg (N=516)** | **Baricitinib 4 mg (N=510)** |
| Study/treatment discontinuation | 117 (23.0) | 60 (11.6) | 117 (22.9) |
| Due to COVID related reasons | 6 (1.2) | 6 (1.2) | 12 (2.4) |
| Not due to COVID related reasons | 111 (21.8) | 104 (20.2) | 105 (20.6) |
| Missing data | 104 (20.4) | 90 (17.4) | 107 (21.0) |
| Due to COVID related reasons | 6 (1.2) | 8 (1.6) | 13 (2.5) |
| Not due to COVID related reasons | 98 (19.3) | 82 (15.9) | 94 (18.4) |
| Prohibited medication increase/initiation | 33 (6.5) | 22 (4.3) | 23 (4.5) |
| SLEDAI not reduced by >=4 points | 159 (31.2) | 165 (32.0) | 128 (25.1) |
| BILAG criteria not met | 11 (2.2) | 10 (1.9) | 7 (1.4) |
| PGA criteria not met | 9 (1.8) | 9 (1.7) | 2 (0.4) |
